# The effectiveness of real‐time identification of medication ingestion in schizophrenic patients in Taiwan

**DOI:** 10.1002/kjm2.12637

**Published:** 2022-12-19

**Authors:** Chin‐Yin Chen, Tyng‐Yeu Liang, Chih‐Hung Ko, Hsiu‐Fen Hsieh

**Affiliations:** ^1^ Nursing Department Kaohsiung Chang Gung Memorial Hospital Kaohsiung Taiwan; ^2^ Department of Electrical Engineering National Kaohsiung University of Science and Technology Kaohsiung Taiwan; ^3^ Department of Psychiatry Kaohsiung Municipal Hsiao‐Kang Hospital Kaohsiung Taiwan; ^4^ College of Medicine Kaohsiung Medical University Kaohsiung Taiwan; ^5^ College of Nursing Kaohsiung Medical University Kaohsiung Taiwan; ^6^ Department of Nursing Kaohsiung Medical University Hospital Kaohsiung Taiwan; ^7^ Department of Medical Research Kaohsiung Medical University Hospital Kaohsiung Taiwan

Schizophrenia is a mental disorder that causes severe functional disabilities and imposes a heavy economic burden on patients and their families. Poor medication adherence is a major cause of relapse of psychiatric symptoms in schizophrenic patients.^1^ Anti‐psychotic drugs are the first choice for alleviating the psychiatric symptoms of schizophrenic patients, and the goal of regular medication is to prevent recurrence and overall functional deterioration.^2^ The efficacy of anti‐psychotic drugs in improving the psychiatric symptoms of schizophrenic patients has been verified, but it remains common for schizophrenic patients to not adhere to their prescriptions. This is caused by multiple reasons: stigma of mental illness, drug side‐effects, forgetting to take medicine, lack of insight, and so forth.^3^ Previous research has indicated that the proportion of medication nonadherence among patients who are diagnosed with schizophrenia for the first time is as high as 50% in the first year.^4^

Schizophrenic patients are interested in utilizing innovative tools, and the application of electronic health record technology, such as wearable devices, smartphone apps, telepsychiatry, and patient communication systems in remote areas can improve access to mental health services and alleviate psychiatric symptoms for schizophrenic patients.^5^ The MedAdhere app was designed and developed by the authors' research team, and this app can be downloaded in the Google Play Store at https://play.google.com/store/apps/developer?id=NKUST-HPDS. The researcher downloaded this app for each participant and assisted them to set up its functions, such as recognition of patient's face and each of his/her anti‐psychotic medications, dose and time for medication, and so forth. Figure 1 showed the steps of anti‐psychotic ingestion by patients and the processes of recognition by MedAdhere app. Each step will be real‐time recorded and uploaded to the cloud server. This app can also be used offline and it can automatically upload all records to the cloud server when connecting to an internet network, allowing the researchers to observe a patient's medication status. The data were collected between June 2021 and February 2022. A total 0f 20 participants in a psychiatric day‐care centre at a medical centre completed all the processes of this study.

**FIGURE 1:**
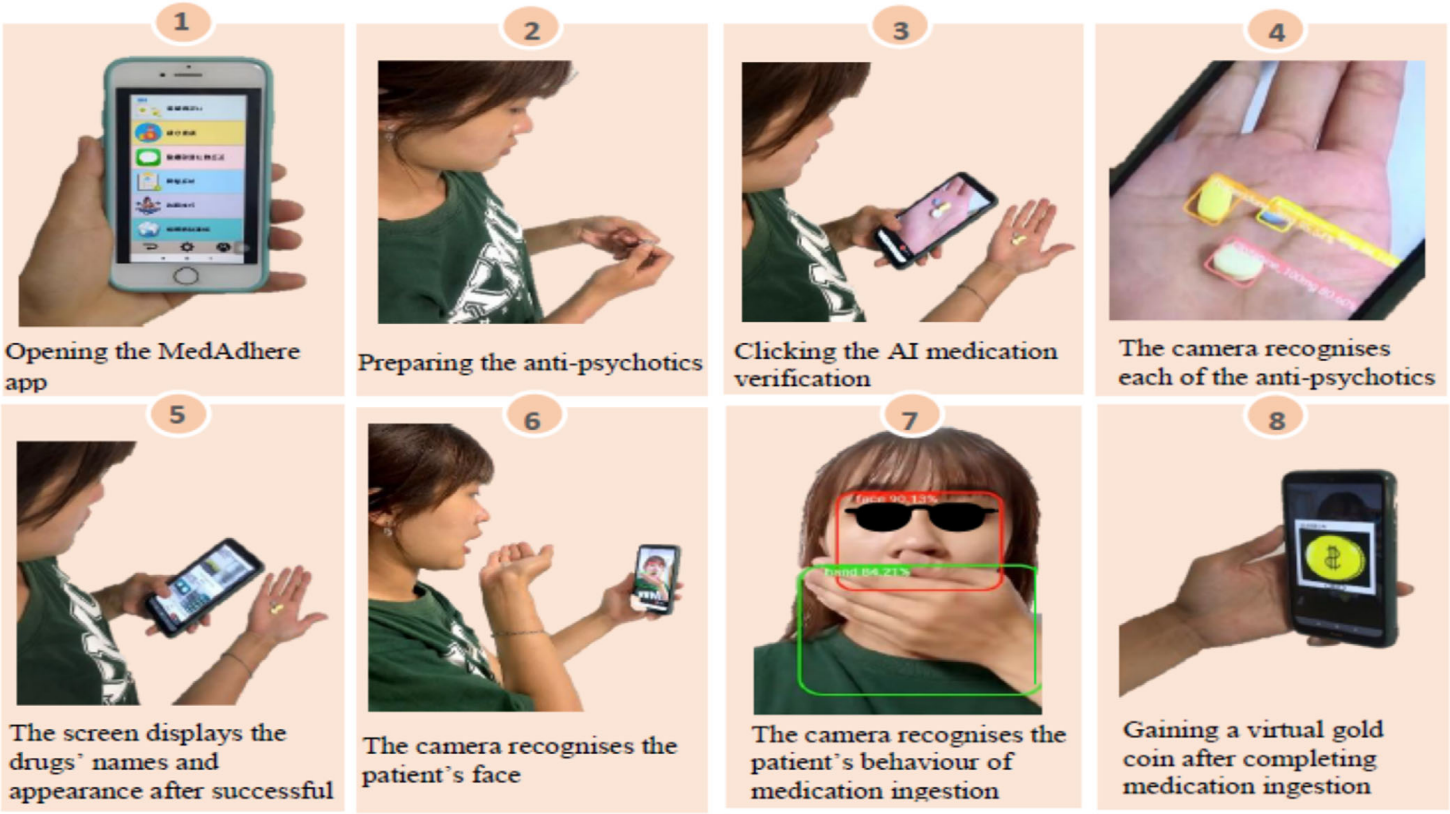
Steps of anti‐psychotic ingestion recognized by MedAdhere app

Medication adherence of our participants was 90% during the intervention. The positive and negative syndrome scale showed that participants' positive symptoms, negative symptoms, and general psychopathology symptoms significantly improved after intervention. These precisely auxiliary functions of the MedAdhere App were based on AI technology and our results suggested that schizophrenic patients' medication adherence can be further enhanced by the application of such AI technology. This MedAdhere app can be introduced to other countries and translated into its native language since the prevalence of schizophrenic disorder is similar between countries and imperfect medication adherence is a universal phenomenon among schizophrenic patients. Moreover, using this MedAdhere app, the healthcare providers do not need to contact the patients, and this further reduces the risk of mutual infection, it's another advantage, particularly important in the context of COVID‐19.

## CONFLICT OF INTEREST

All authors declare no conflict of interest.
